# Temporal Trajectories of Depressive Symptoms, Apathy, ADL and Cognitive Decline in Older People; a Dynamic Time Warping network analysis

**DOI:** 10.1192/j.eurpsy.2025.291

**Published:** 2025-08-26

**Authors:** A. J. C. Van Der Slot, S. P. Mooijaart, J.-W. Van Dalen, M. P. Hoevenaar, E. Richard, E. J. Giltay

**Affiliations:** 1 Psychiatry, LUMC; 2 Internal Medicine, section of Gerontology and Geriatrics, Leiden University Medical Center, Leiden; 3 Neurology, Amsterdam UMC, Amsterdam; 4 Neurology, Amsterdam UMC, Leiden; 5 Neurology, RaboudUMC, Nijmegen; 6 Public Health & Primary Care, Health Campus The Hague, The Hague, Netherlands

## Abstract

**Introduction:**

The prevalence of depressive symptoms and cognitive decline increases with age. Understanding the temporal dynamics of these symptoms could provide valuable insights into the early stages of cognitive decline, allowing for more timely and effective treatment and management.

**Objectives:**

Our objective was to explore how depressive symptoms, apathy, limitations in daily life activities, and cognitive impairment evolve and interact over time in older individuals. Specifically, we aimed to determine whether changes in these symptoms could help identify subsequent cognitive decline. We used Dynamic Time Warping analysis to model and characterize the progression of these symptoms, examining their relationships both at individual and group levels.

**Methods:**

Participants from the Prevention of Dementia by Intensive Vascular Care (preDIVA) trial cohort with baseline and ≥3 follow-up measurements were included, with a median of 6.7 years of follow-up. Dynamic Time Warping analysis was used to model temporal dynamics of individual constituents of the Mini Mental State Exam (MMSE), activities of daily living (ADL) using the Amsterdam Linear Disability Scale (ALDS) and depressive symptoms using the 15-item Geriatric Depression Scale (GDS-15).

**Results:**

The 1537 participants had an average age of 74 years at baseline, 56.5% were female, and 19.9% had finished a higher education. The directed analyses revealed a nuanced temporal pattern, wherein certain depressive symptoms preceded cognitive decline indicators, and vice versa. The GDS-15 symptoms with the strongest outstrength, meaning changes in these symptoms preceded subsequent changes in other items, were the apathy symptoms ‘dropped activities/interests’, ‘energetic’ and ‘not doing new things’ (all p’s<.001). The MMSE constituents with the strongest instrength, meaning changes in these symptoms were preceded by changes in other items, were ‘immediate memory’, ‘verbal comprehension’ and ‘naming objects’. Decline in ADL function was a consistent predictor of worsening of depressive symptoms and cognitive decline (p<.001).

**Image 1:**

**Figure:**
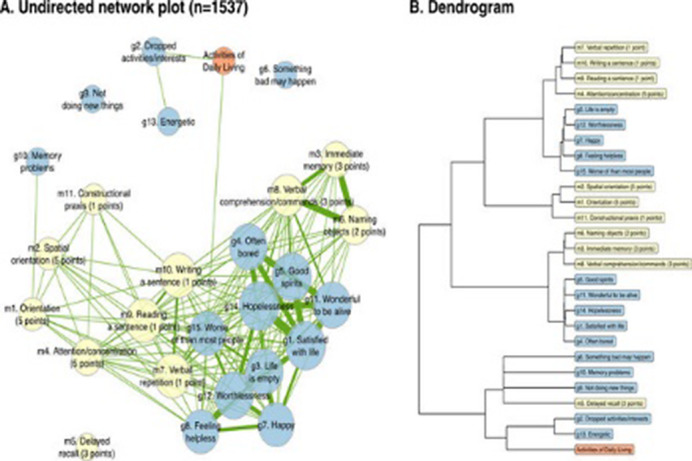

**Image 2:**

**Figure:**
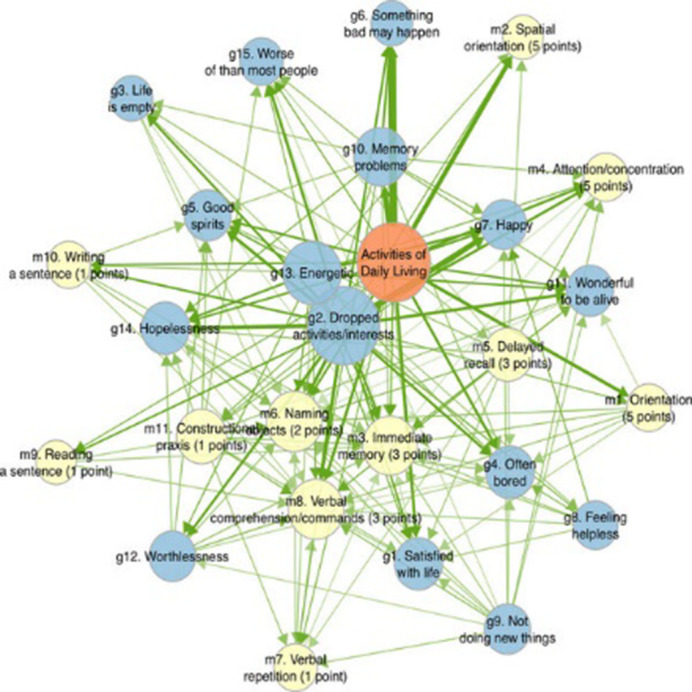

**Image 3:**

**Figure:**
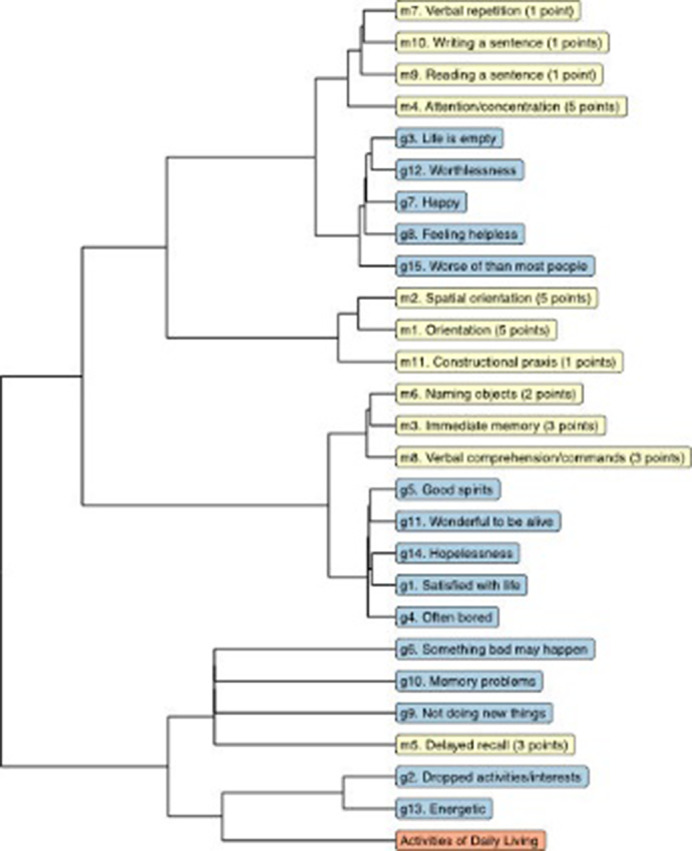

**Conclusions:**

An increase in apathy and a decline in ADL preceded mood-related symptoms and cognitive impairment in older people aged 70-78 years.

**Disclosure of Interest:**

None Declared

